# Comment on “The Incidence of QT Prolongation and Torsades des Pointes in Patients Receiving Droperidol in an Urban Emergency Department”

**DOI:** 10.5811/westjem.2020.9.49486

**Published:** 2021-01-11

**Authors:** Philip C. DiSalvo, Timothy C. Backus, William K. Chiang

**Affiliations:** * Ronald O. Perelman Department of Emergency Medicine, Division of Toxicology, NYU Grossman School of Medicine, New York, New York; † New York City Poison Control Center, Department of Health and Mental Hygiene, New York, New York

Dear Editors:

We read with interest the recent article discussing QT prolongation and torsade des pointes (TdP) and droperidol.^1^ The paucity of readily available antipsychotics and antiemetics that are not associated with QT prolongation makes selection of an appropriate pharmaceutical challenging in ideal situations and decidedly complex when confronted with an agitated, delirious, or intoxicated patient.

Our issue lies with the determination of incidence of TdP. The authors identify one case of TdP among the 16,546 patients who received droperidol. They found this case by examining electrocardiograms (ECG) and computerized ECG reports for 396 and 2431 patients, respectively, and by reviewing an electronic health record for documented diagnoses of TdP, ventricular fibrillation, and ventricular tachycardia. We are concerned that this analysis might have missed two groups of patients.

First, we suspect most patients receiving droperidol are not subject to continuous cardiac monitoring. TdP frequently terminates spontaneously and would likely not be captured on a single post-administration ECG, even in the limited subset for whom one was done.^2^ In an (intentionally) sedated patient, the episode may go unnoticed and unrecorded. Nor would a transient episode in an alert patient likely be recorded as one of the diagnoses the authors queried, but instead as lightheadedness, syncope, palpitations, chest pain, seizure, or any number of subjective complaints. While these episodes may be less clinically relevant than an episode of TdP that degenerates into ventricular fibrillation, they nonetheless reflect real and important contributions to the incidence.

Secondly, we are told little of the disposition of the patients under study. How many of these patients were discharged from the emergency department (ED) after receipt of droperidol? How long were they observed before discharge? While the risk of QT prolongation may be highest in the initial period after administration, the absolute duration of risk is not known, particularly with the wide range of doses that are described in clinical practice. The authors describe a patient population with high rates of alcohol and/or substance use disorders – a population that is at persistent risk of exposure to another QT prolonging xenobiotic after discharge, either in the form of a drug of abuse or iatrogenically upon presentation to a different ED.

A rigorous calculation of incidence typically requires a prospective study. In this case, one might be more reassured by a study in which the cohort of interest undergoes ECG to confirm normal QT before exposure, receives a standardized dose of droperidol, and then undergoes serial ECGs and continuous cardiac monitoring for the expected duration of effect. We acknowledge that recruitment of a sufficiently large population to measure a rare adverse event would be challenging, but we see no alternative to calculating an accurate incidence.

We appreciate the efforts of Dr. Cole et al. and largely agree with their thoughtful discussion of the limited evidence upon which droperidol’s black box warning was issued. Droperidol may in reality have a low risk of TdP in comparison to other antiemetics or antipsychotics, but we would like to caution readers from relying on this methodology to make determinations of the incidence of rare adverse events, or to find assurances of safety.
